# A Biodiversity Indicators Dashboard: Addressing Challenges to Monitoring Progress towards the Aichi Biodiversity Targets Using Disaggregated Global Data

**DOI:** 10.1371/journal.pone.0112046

**Published:** 2014-11-19

**Authors:** Xuemei Han, Regan L. Smyth, Bruce E. Young, Thomas M. Brooks, Alexandra Sánchez de Lozada, Philip Bubb, Stuart H. M. Butchart, Frank W. Larsen, Healy Hamilton, Matthew C. Hansen, Will R. Turner

**Affiliations:** 1 NatureServe, Arlington, Virginia, United States of America; 2 Department of Environmental Science and Policy, George Mason University, Fairfax, Virginia, United States of America; 3 International Union for Conservation of Nature, Gland, Switzerland; 4 World Agroforestry Center, International Center for Research in Agroforestry, University of Philippines, Los Baños, Laguna, Philippines; 5 School of Geography and Environmental Studies, University of Tasmania, Hobart, Australia; 6 United Nations Environment Programme World Conservation Monitoring Centre, Cambridge, United Kingdom; 7 BirdLife International, Cambridge, United Kingdom; 8 European Environment Agency, Copenhagen, Denmark; 9 Conservation International, Arlington, Virginia, United States of America; 10 Department of Geographical Sciences, University of Maryland, College Park, Maryland, United States of America; Oregon State University, United States of America

## Abstract

Recognizing the imperiled status of biodiversity and its benefit to human well-being, the world's governments committed in 2010 to take effective and urgent action to halt biodiversity loss through the Convention on Biological Diversity's “Aichi Targets”. These targets, and many conservation programs, require monitoring to assess progress toward specific goals. However, comprehensive and easily understood information on biodiversity trends at appropriate spatial scales is often not available to the policy makers, managers, and scientists who require it. We surveyed conservation stakeholders in three geographically diverse regions of critical biodiversity concern (the Tropical Andes, the African Great Lakes, and the Greater Mekong) and found high demand for biodiversity indicator information but uneven availability. To begin to address this need, we present a biodiversity “dashboard” – a visualization of biodiversity indicators designed to enable tracking of biodiversity and conservation performance data in a clear, user-friendly format. This builds on previous, more conceptual, indicator work to create an operationalized online interface communicating multiple indicators at multiple spatial scales. We structured this dashboard around the Pressure-State-Response-Benefit framework, selecting four indicators to measure pressure on biodiversity (deforestation rate), state of species (Red List Index), conservation response (protection of key biodiversity areas), and benefits to human populations (freshwater provision). Disaggregating global data, we present dashboard maps and graphics for the three regions surveyed and their component countries. These visualizations provide charts showing regional and national trends and lay the foundation for a web-enabled, interactive biodiversity indicators dashboard. This new tool can help track progress toward the Aichi Targets, support national monitoring and reporting, and inform outcome-based policy-making for the protection of natural resources.

## Introduction

Resource monitoring has long been recognized as a cornerstone of biodiversity and conservation science [1], [2], [3]. In 2010, at the 10^th^ Conference of the Parties of the Convention on Biological Diversity (CBD), 193 nations agreed to twenty “Aichi Biodiversity Targets”, and in doing so committed to updating their National Biodiversity Strategies and Action Plans and developing monitoring programs to assess progress [4]. The Aichi Targets rely upon indicators to report progress towards reducing pressure on biodiversity, maintaining and improving the state of biodiversity, implementing conservation actions to ameliorate biodiversity loss, and providing benefits to human well-being [4]. Many other initiatives and multilateral agreements call for similar indicator-based biodiversity monitoring, including (a) the United Nations Millennium Development Goal #7 [5] and the draft new Sustainable Development Goals [6]; (b) intergovernmental treaties that provide mechanisms for national action and international cooperation, such as the Ramsar Convention on Wetlands [7] and the Convention on Migratory Species [8], (c) science-policy interfaces such as the Intergovernmental Platform on Biodiversity and Ecosystem Services [9]; and (d) partnerships or networks in support of the above mentioned bodies, such as the Biodiversity Indicators Partnership [10], [11], [12], and the Group on Earth Observations Biodiversity Observations Network Working Group #9 [13].

Monitoring called for by these programs is essential both to document biodiversity change over time [14], to shed light onto key ecological processes [15], and to measure the success or failure of conservation interventions through counterfactual analysis [16], [17], [18], [19]. However, most existing monitoring programs have been designed primarily at localized scales, and often produce information that is disaggregated, heterogeneous, and non-standardized when considered at national or regional scales [20]. Monitoring requirements for measuring conservation performance, of the kind necessary to track the Aichi Targets, require data that transcend the fine temporal, spatial, and organizational scales commonly addressed in current literature [15].

Documentation of conservation impacts and biodiversity response must be accomplished in ways that are scientifically defensible, at appropriate temporal and spatial scales, and simple enough to inform decision-making by the diverse group of individuals and organizations working at the intersection of science and policy. Mounting global evidence shows that biodiversity loss is continuing at alarming rates [21], [22], yet currently, two thirds of national reports submitted to the CBD lack evidence-based measures to illustrate changes in the status of biodiversity [23]. National capacity is often insufficient to measure many indicators of interest using on-the-ground methods, particularly in developing countries [24]. Even when national data are available, a lack of standardization across countries can make regional assessment difficult or impossible [20].

To better understand the challenges to effective biodiversity monitoring at national and regional scales, and how finer-scale (e.g. national) data might be integrated into a framework for global monitoring of biodiversity status and trends, we surveyed local conservation experts working in areas of high conservation value on monitoring and capacity needs. Building from the needs identified in those workshops, we then developed the concept for a biodiversity indicators dashboard using indicators derived from global data sets and constructed a dashboard prototype. This is the first operationalized dashboard to date that communicates multiple biodiversity indicators at multiple scales, and directly serves the global need to monitor progress towards Aichi Targets. Full development of the biodiversity indicators dashboard will encompass: (1) identification of appropriate indicators, (2) proof of concept using global data, (3) building the technological infrastructure necessary to host the dashboard, (4) designing the visual interface for multiple platforms (i.e. web and mobile users), and (5) creating systems to support the integration of finer-scale (regional and national) data. Here, we address in detail steps 1 and 2 of the dashboard design, laying the foundation for a web-based tool freely available to all with an interest in biodiversity conservation. A prototype of the tool is now available to the international conservation community at http://dashboarddev.natureserve.org, with steps 3–5 being implemented in an on-going iterative process.

## Methods and Results

### 1. Study Area

**Figure 1:**
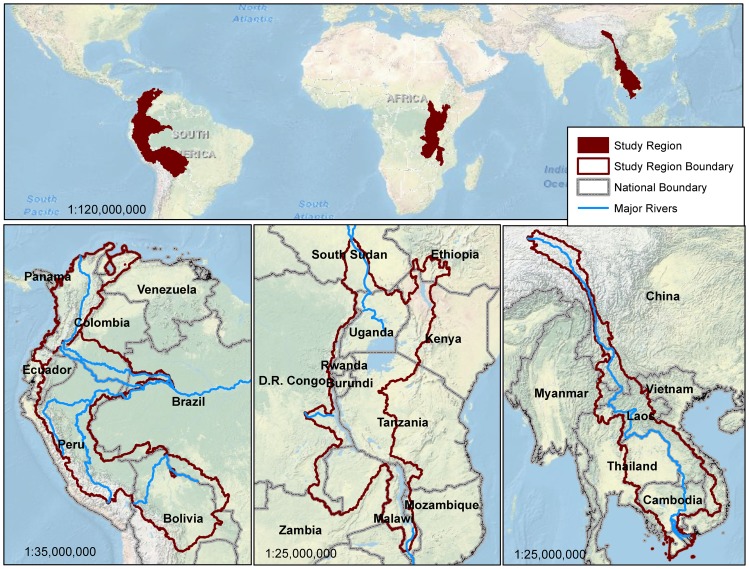
Study area regions. From left to right: the Tropical Andes, the African Great Lakes, and the Greater Mekong.

We considered three geographically diverse areas with exceptional biodiversity value, that confront a high degree threat and that receive significant investment by international conservation agencies (Figure 1) [25], [26]. The Tropical Andes region encompasses the eastern slope of the Andes, containing eight watersheds of headwater rivers (Japura, Putumayo, Rio Maranon, Ucayali, Guapore, Madre de Dios/Beni, Amazon, Magdalena) across Venezuela, Colombia, Ecuador, Peru, and Bolivia. The Great Lakes region of Africa includes five major watersheds (Lake Victoria, Upper Nile, Lake Tanganyika, Lake Malawi/Nyasa, Turkana/Omo) across Ethiopia, South Sudan, Kenya, Uganda, Democratic Republic of Congo, Rwanda, Burundi, Tanzania, Zambia, Malawi, and Mozambique. The Greater Mekong region encompasses the entire Mekong River Basin, spanning China, Myanmar, Vietnam, Lao P.D.R., Cambodia, and Thailand [25].

We delineated regional boundaries for the Tropical Andes, African Great Lakes and Greater Mekong regions using hydrological basins derived from HydroSHEDS and compiled by the UN-FAO, [27], [28], [29]. We performed analyses at both this regional scale, and at the national scale for the 22 countries that these three regions overlap (including areas outside the focal watershed boundaries).

### 2. Challenges to Biodiversity Monitoring and Capacity Needs at Regional and National Scales

We conducted seven consultation workshops in the three study regions between September 2011 and August 2012 to (1) better understand the challenges to effective biodiversity monitoring at national and regional scales, (2) identify gaps in current monitoring capacity and potential mechanisms for filling those gaps, and (3) begin to explore mechanisms for integrating local and national monitoring data into future regional and national biodiversity indicators. In total, 260 individuals from 20 countries attended at least one of the workshops, with broad representation from the public, civil-society, and academic sectors. Invitees included those with professional responsibility for National Biodiversity Strategies and Action Plans for monitoring progress towards Aichi Targets, and managers and technical experts responsible for designing and conducting biodiversity monitoring programs at multiple scales.

At each workshop, we solicited multiple-choice feedback on two issues: 1) the spatial scales of monitoring that participants required to guide their work (regional, national, sub-national, watershed, and/or site scales); and 2) the status of monitoring of selected biodiversity indicators for pressure, state, response, and benefits at the national scale, with answer options of “Monitored”, “Limited Monitoring” (monitoring that has been conducted in some areas but not systematically done across the country), “Not Monitored”, or “Unknown”. Of the 260 workshop participants, 132 (51%) submitted answers to these written questionnaires, of which 39% came from the public sector, 45% from civil-society, and 16% from the academic sector. We also recorded and categorized responses to open-ended questions addressing (1) the utility of tracking biodiversity indicators derived from existing global data with a dashboard approach and (2) national challenges in developing sustainable biodiversity monitoring.

To identify the preferred scales of monitoring, we tabulated the frequency of the scales that participants indicated were important. To quantify the existing capacity for monitoring in each of the targeted countries, we calculated a score based on the perceived monitoring status for each biodiversity indicator. The score is scaled 0 (not monitored) to 1 (monitored), and equals *P_1_* + 0.5*P_2_*, with *P_1_* the percent of respondents who answered “monitored” and *P_2_* the percent of respondents who answered “limited monitoring”. We used ANOVA to explore differences of monitoring status between regions, and a repeated-measures ANOVA to examine differences in monitoring status among indicators.

Responses to the questionnaire indicate a strong demand for reliable information on the state of, and pressures facing, biodiversity. Regarding scales of monitoring, participants were most interested in analyses carried out at the site (82%) and national levels (76%), followed by watershed (71%), sub-national (68%) and regional levels (65%).

**Figure 2:**
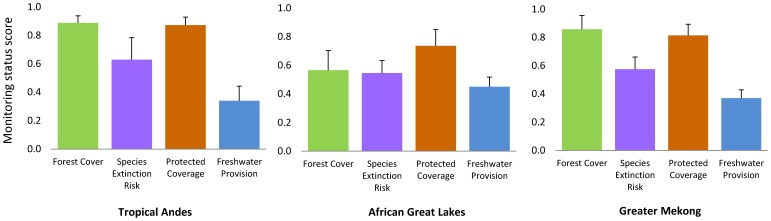
Monitoring status of the indicators, as reported by national experts via questionnaire responses. The mean score and its standard error for each indicator are shown by region. Number of respondent is 36 for Tropical Andes, 46 for African Great Lakes, and 50 for Greater Mekong.

Our questionnaires revealed significant differences in the degree to which indicators are currently monitored (p<0.001), with hydrologic measures (average score  = 0.40) and species extinction risk (0.57) less frequently monitored than deforestation (0.72) and protected area coverage (0.79) (Figure 2). While there were no differences in the average score across regions (p = 0.88), the status of monitoring differed widely among nations. Of the 22 countries, those with the highest overall scores for existing monitoring were Colombia (0.875), Malawi (0.875), and Thailand (0.75). Countries with very limited monitoring include South Sudan and the D. R. Congo (both ≤0.25).

**Figure 3:**
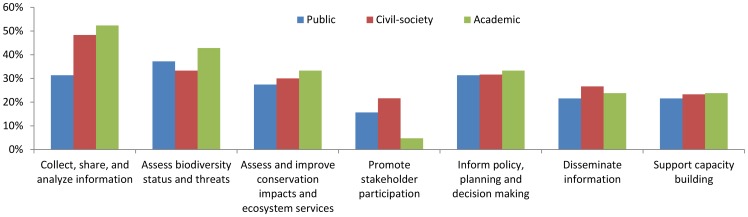
Perceived benefits of using global data within a dashboard approach, by sector. Number of respondent is 51 for public sector, 60 for civil-society, and 21 for academic sector.

Among the open-ended questions, a third of survey respondents from all sectors expressed high interest in using the dashboard approach, and employing appropriate subsets of global scale data, as a means to gather and share information to assess biodiversity status and threats, assess and improve conservation impacts, and inform policy, planning, and decision-making. Supporting capacity building, promoting stakeholder participation and dissemination of information were also frequently cited by survey respondents as potential benefits of this effort (Figure 3).

**Figure 4:**
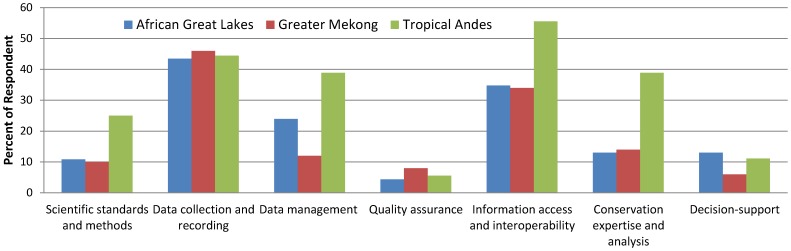
Perceived challenges to biodiversity monitoring by region. Number of respondent is 36 for Tropical Andes, 46 for African Great Lakes, and 50 for Greater Mekong.

Across regions, the challenges to effective monitoring (Figure 4) include the lack of personnel, technology, and financial support for data collection and management (45%), and limited information accessibility and interoperability (40%). Emphasis varies among regions, with African respondents stressing the need for support in data management (24%), and Andean respondents more concerned about scientific standards and methods (25%) and conservation expertise and analysis (39%).

### 3. Creation of a Biodiversity Indicators Dashboard to Support Monitoring Needs

#### 3.1 The Dashboard Concept

To address the challenges to biodiversity monitoring at regional and national scales identified by the survey, we envision the creation of a biodiversity “dashboard” – a visualization of biodiversity indicators designed to enable ongoing tracking of biodiversity status and trends and present biodiversity monitoring and conservation performance information in a clear, user-friendly, and unified format that facilitates iterative adaptive management. Using biodiversity indicators of the type developed by Butchart et al. (2010) [22] as the foundation, the dashboard provides a means to disseminate information, promote stakeholder participation, support capacity building, and allow users to better understand the relationship between conservations actions and impact. The utility of the biodiversity indicators dashboard in meeting these needs was confirmed by responses to our survey (Figure 3).

Originating as a business performance monitoring tool, dashboard visualizations are an information management and reporting instrument that has seen increasing use in a variety of contexts to communicate complicated information on current status and historical trends to broad audiences [30], [31]. From the World Bank Atlas of Global Development [32] to commercial products used to track stock performance and guide financial investment (e.g., [33]), dashboards distill complicated data by tracking key indicators, usually via a combination of charts and maps. This information is typically served on websites or mobile applications and updated regularly (e.g., annually for World Bank indicators, minute-by-minute for financial markets).

Dashboards have been proposed and employed in various biological and resource management contexts. For example, the CITES Trade Data Dashboard [34] allows users to explore patterns in species exploitation across space, time, and taxonomic affiliation through a dynamic interface [35]. Dashboards also support fisheries management by providing a framework to better visualize relationships among fish populations, socio-economics, and exploitation [36], [37].

If a dashboard is to be useful for decision makers, the indicators chosen must present information critical to influencing the decisions to be made. We used the Pressure-State-Response-Benefit (PSRB) framework to guide selection of indicators, following Sparks et al. 2011 [14]. This is derived from the causal-chain Pressure-State-Response and Driver-Pressure-State-Impact-Response frameworks, widely used for reporting on the state of the environment [22], [23], [38], [39], [40], [41], [42], [43], and one that has been used by the CBD Ad-Hoc Technical Expert Group of the CBD to guide indicator development for the CBD [44] and recommended for communicating biodiversity indicators [42]. The core elements in PSRB as applied in the dashboard assessments are pressure on biodiversity, its drivers, (e.g., habitat destruction, climate change, invasive species), the state of species and ecosystems (e.g., species extinction risk, animal and plant populations, ecosystem integrity), conservation action or policy responses (e.g., protected areas establishment and management, investment in biodiversity conservation) and benefit to human well-being from the social, economic and cultural impacts of conservation (e.g., maintenance of hydrological functions, climate change mitigation, maintenance of indigenous cultures). By viewing dashboard indicators together within the PSRB framework, users can begin to understand interactions between indicators [14].

#### 3.2 Methods for Constructing a Biodiversity Indicators Dashboard using Global Data

We selected one indicator from each PSRB component, with consideration for the availability of global datasets, the degree to which the indicator contributes to evaluating progress made towards the Aichi Targets, the feasibility of trend estimates, and the likely availability of analogous data generated locally for future integration into the dashboard. The four selected indicators are examples of the types of data the biodiversity indicators dashboard can be used to track. At this stage in development of the dashboard, the chosen indicators are not intended to address causal relationships; however, as additional indicators are added, the PSRB approach will facilitate exploration of causal links between indicators. We selected forest cover loss as the pressure indicator, species extinction risk as the state indicator, protected area coverage of key biodiversity areas (KBAs) as the response indicator, and freshwater provisioning to downstream human populations as the benefit indicator. We used data from the first decade of the 21^st^ century to represent current status and provide an initial baseline, or reference point, against which future trends can be assessed. For all but the benefit indicator (freshwater provision), existing data from either previous time steps (i.e. species extinction risk and percent protection of KBAs) or later time steps (i.e. forest cover loss) supporting the tracking of trends.

Global data were disaggregated to provide regional and national indicator values (Table 1). For each indicator, we mapped current condition, charted and mapped trends over time, and generated tabular summaries. All spatial analyses were performed using ArcGIS 10.1 [45] and all statistical calculations were performed in R [46].

**Table 1:** Biodiversity indicators summary and data sources.

Framework Component	Pressure/Driver	State	Response	Benefit (Impact)
Indicator	Forest coverage and rate of gross forest cover loss	Red List Index	Protected area coverage in key biodiversity areas	Quality-weighted freshwater provision from natural ecosystems to downstream human population
Aichi Target	Target 5: Loss of habitats is at least halved by 2020	Target 12: Extinctions of known threatened species has been prevented by 2020	Target 11: At least 17% of terrestrial…areas, especially important areas for biodiversity and ecosystem services are conserved by 2020	Target 14: Ecosystems that provide essential services are restored and safeguarded by 2020
What does it show	Spatial data represents the percent of forest cover for each 18.5 km pixel in 2000 and percent gross forest cover loss (i.e., deforestation) from 2000 to2005. Tabular FAO data summarize forest land use coverage and "net forest cover change" by country in 2005 and 2010.	An index of aggregate survival probability of species that occur in the given spatial unit. Values range from 1 (all species Least Concern) to 0 (all Extinct).	Mean percent area of key biodiversity areas covered by protected areas	Quality-weighted delivery of clean freshwater from natural habitats to downstream human populations per unit area
Data Source	Forest cover for year 2000 and gross forest cover loss 2000–2005 through Global Forest Monitoring Project	IUCN Red List assessment for:	- World Database on Protected Areas (UNEP-WCMC) (2010)	- World WaterGAP 2 model runoff map
				- Hydrological drainage direction
		- Amphibians (1980, 2004)	- Global KBAs as represented by Important Bird and Biodiversity Areas (IBAs) and Alliance for Zero Extinction (AZE) sites	
				- Landscan Global Population Database
		- Birds (1988, 2008)		
				- GlobCover land cover
		- Mammals (1996, 2008)		
Time frame	2000–2005	1980–2008	1950–2010	2010
Limitation and caveat	- Resolution is too coarse (18.5 km) to detect deforestation in small areas.	- Differing assessment dates requires interpolation and extrapolation to estimate aggregate trends	- The WDPA omit recently decreed protected areas	- Values are relative, not absolute
	- The gross forest cover loss data shows deforestation only, not taking account afforestation	- Because of the heterogeneous distribution of species, regional extinction risk can skew national indicator values	- The WDPA does not currently document management effectiveness	- Only baseline (2010) data currently available; not able to estimate trend
	- Forest degradation was not quantified	- The proportion of a species' range within a given analysis unit is not considered	- Key biodiversity areas for taxa other than birds that are not endemic to single sites have only been identified in some countries	- Spatial resolution is too coarse (2,5921km^2^ pixels) to estimate freshwater provision in small areas
		- Red List categories are necessarily broad classes of extinction risk, so the RLI is moderately sensitive		

a. Pressure Indicator: Forest Loss

The forest loss indicator is derived from the Global Forest Monitoring Project [47], [48], which estimated forest cover in 2000 and forest cover loss between 2000 and 2005 using MODIS data [49] calibrated with Landsat [50] imagery, at 18.5-km resolution. Values represent the percent forest cover within each pixel, with forest cover defined as areas with at least 25% cover of trees at least 5 meter in height. The deforestation measure, Gross Forest Cover Loss (GFCL), represents a unidirectional change in forest cover, calculated from the percent forest loss between 2000 and 2005. For each analysis unit (e.g., region, nation) we calculated a mean value for forest cover in 2000 and mean GFCL between 2000 and 2005. We then derived the average annual rate of GFCL for 2000–2005 for each analysis unit, presented as the annual percent forest loss from the 2000 baseline.

While, to our knowledge, the GFCL data provide the best globally consistent spatial representation of deforestation to date, the data are limited in that they do not incorporate information on forest gain from restoration, natural regrowth, and plantation. They also do not address finer resolution forest degradation, as some other regional mapping products do [51], [52], [53].

b. State Indicator: Species Extinction Risk

The Red List Index (RLI) is a measure of trends in survival probability (the inverse of extinction risk) for sets of species. It is based on the numbers of species within each IUCN Red List category (i.e., Extinct (EX), Extinct in the Wild (EW), Critically Endangered (CR), Endangered (EN), Vulnerable (VU), Near Threatened (NT), Least Concern (LC)) and the changes in these numbers over time resulting from genuine improvement or deterioration in status between assessments [22], [54], [55], [56], [57], [58].

For this indicator, we used the first and last comprehensive Red List assessment (when all species of a taxonomic group were assessed) for each of three vertebrate groups (1988 and 2008 for birds, 1996 and 2008 for mammals, and 1980 and 2004 for amphibians, noting that the 1980 assessment for amphibians was based on a retrospective assessment), following Butchart et al. (2004, 2005, 2007 and 2010) [22], [54], [55], [56]. We identified all species falling partially or completely within each region and each country using 2010 spatial distribution data for each species [58] (Table 2). For each region and country, we calculated the RLI for each taxonomic group individually and for all taxonomic groups together. This standardized RLI varies between 1 (all species LC) and 0 (all species EX or EW). Following Butchart et al. 2004, 2005 [55], [56], species undergoing genuine Red List category changes between assessments contributed to RLI trends only if the driving process of the change (i.e. threat or conservation action) operated within the relevant country or region. For each vertebrate group, we calculated the annual change in aggregate extinction risk by dividing the difference in RLI between the last and first assessment by the number of intervening years. Data Deficient and Not Evaluated species were excluded from this calculation and the annual change value across all taxonomic groups is computed using the mean time difference between assessments for the three groups.

**Table 2:** Number of species recorded and analyzed to derive Red List Index (only extant species that are not Data Deficient were included).

	Number of all assessed species				Number of species with changed Red List category			
	Overall	Mammals	Birds	Amphibians	Overall	Mammals	Birds	Amphibians
Assessment year	2008	2008	2008	2004	1980–2008	1996–2008	1988–2008	1980–2004
Tropical Andes	5357	880	2978	1499	179	13	29	137
African Great Lakes	2483	613	1534	336	30	10	17	3
Greater Mekong	2499	642	1479	378	142	41	71	30

c. Response Indicator: Protected Area Coverage of Key Biodiversity Areas

For the Response indicator, we calculated the mean percentage area of key biodiversity areas (sites contributing significantly to the global persistence of biodiversity [59]) falling within protected areas for each analysis unit [60]. We used the World Database on Protected Areas for 2010 [61] to delimit protected areas. Within our study area, key biodiversity areas include 757 Important Bird & Biodiversity Areas (IBAs; the subset of key biodiversity areas important for birds[62]), and 139 Alliance for Zero Extinction sites (AZE; the subset of key biodiversity areas holding effectively the entire populations of highly threatened species, i.e., CR and EN species>95% restricted to single sites [63], [64]). We calculated the percentage of each KBA that overlaps protected area boundaries and subsequently generated a national mean. We used the year of establishment to generate time series graphs for each year from 1950 to 2010, assigning an establishment date to those protected areas lacking establishment date by randomly sampling from known dates of designation of protected areas in the same country, and then bootstrapping following the methods of Butchart et al. [60]. We plotted the mean with 95% confidence intervals based on uncertainty arising from the missing data [60]. We also calculated the annual rate of change in protection of key biodiversity areas between 1980 and 2010. The annual rate of change is thus from a time period comparable to that calculated for the State indicator (the Red List Index).

d. Benefit Indicator: Freshwater Provision

Freshwater provision data were developed by Larsen et al. [65], [66] using spatially explicit maps of runoff from the global hydrological water model WaterGAP [67], hydrological drainage directions [27], [68], downstream human population density [69], and global land cover data (used to weight flow estimates by a quality coefficient, based on information from previous studies [70], [71], [72], [73]). Estimated quality-weighted freshwater provision, reported as a freshwater flow index, was calculated for 2,592 km^2^ hexagonal grid cells [66]. Using this grid, we calculated a mean value for each analysis unit. Because the freshwater provision data is currently only available for a single time step (2010), we cannot yet calculate trends.

#### 3.3 Results of Biodiversity Indicators Dashboard with Global Data

**Figure 5:**
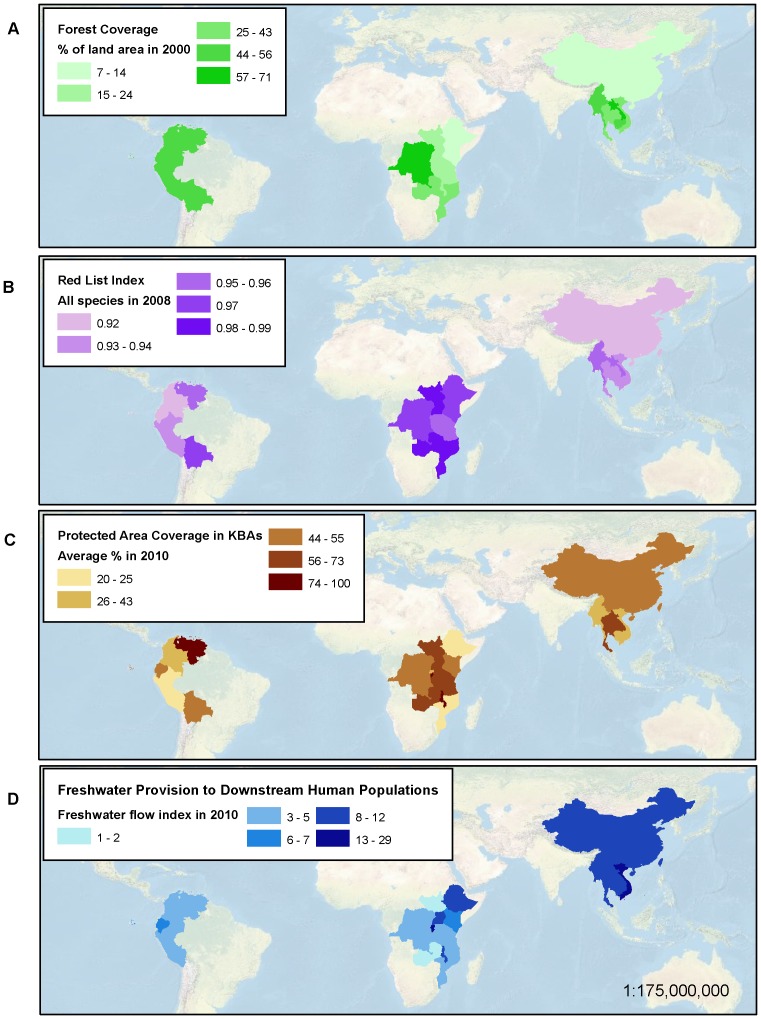
Dashboard indicator baseline results. Results for (A) Forest Cover (2000); (B) Red List Index a measure of change in extinction risk (2008); (C) Protected Area Coverage of Key Biodiversity Areas (2010); and (D) Freshwater Provision (2010).

**Figure 6:**
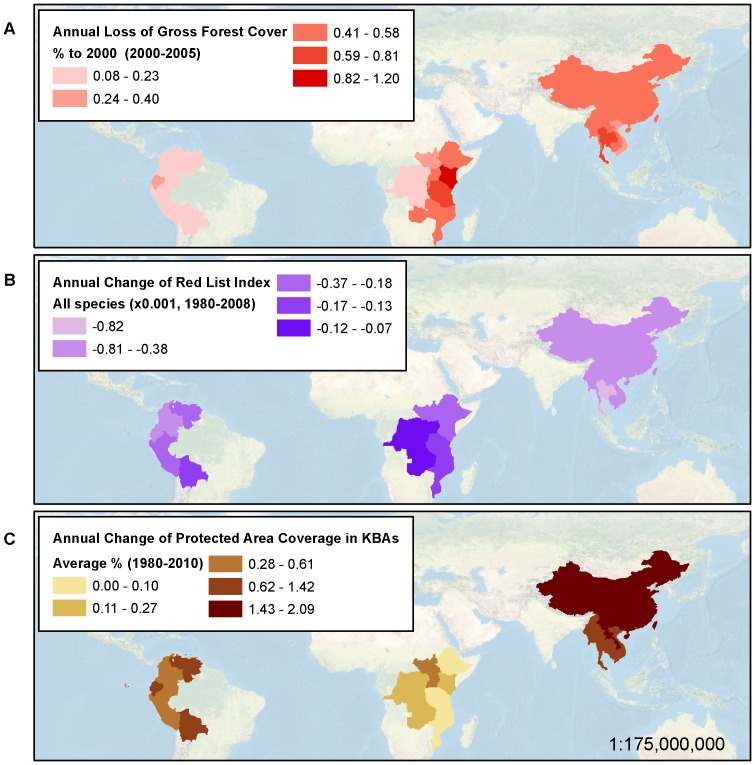
Dashboard indicator trend results. Annual rate of (A) Gross Forest Cover Loss (2000–2005); (B) Change in Red List Index as a measure of extinction risk (change for all species of mammals, birds, and amphibians; 1980–2008); and (C) Change of Protected Area Coverage of Key Biodiversity Areas (1980–2010).

**Figure 7:**
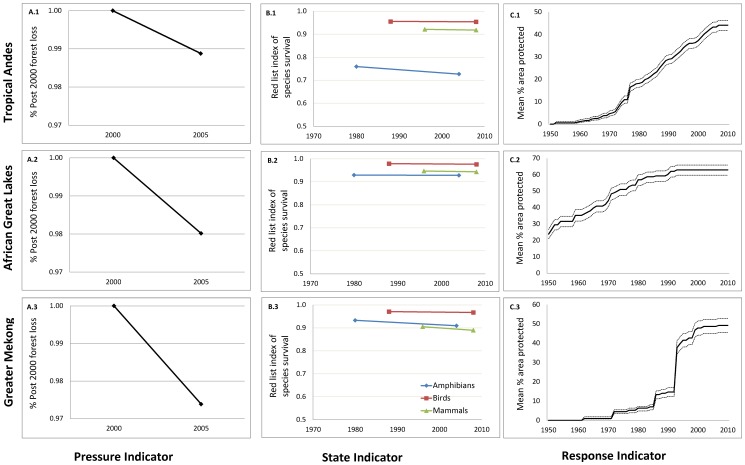
Dashboard indicator trend graphs by region. A.1 – A.3 chart gross forest loss as a percent of forest cover in 2000; B.1-B.3 chart change in Red List Index for mammals (green), birds (red), and amphibians (blue); and C.1-C3 chart change in protected area coverage of key biodiversity areas (1950–2010) with solid lines indicating the mean percent protected across all sites, and dashed line indicating the 95% confidence intervals [60].

The indicators are presented as a series of maps and charts visualized to support a web-enabled biodiversity indicators dashboard (Table 3, Figures 5–7). A web prototype of the dashboard, displaying the results discussed here, is available at http://dashboarddev.natureserve.org.

**Table 3:** Baseline and trend results for all indicators by country and region.

	Indicators – Baseline							Indicators – Annual Rate of Change					
Country/Region	Forest Coverage % (2000)	Red List Index of species survival				Protection Coverage of KBAs % (2010)	Freshwater Flow Index	GFCL % (2000–2005)	Red List Index of species survival (x 0.001)				Protection Coverage of KBAs % (1980–2010)
		3 taxa (2008)	Mammal (2008)	Bird (2008)	Amphibian (2004)				3 taxa (1980–2008)	Mammal (1996–2008)	Bird (1988–2008)	Amphibian (1980–2004)	
Bolivia	56.26	0.97	0.96	0.98	0.90	51.51	1.13	0.21	−0.16	−0.19	−0.06	−0.62	1.03
Colombia	56.06	0.92	0.93	0.97	0.77	42.15	4.82	0.22	−0.38	−0.35	−0.04	−1.09	0.59
Ecuador	50.1	0.92	0.93	0.97	0.73	51.77	6.56	0.29	−0.44	−0.39	−0.03	−1.57	1.24
Peru	52.23	0.94	0.93	0.97	0.82	25.07	2.89	0.08	−0.23	−0.21	−0.05	−0.91	0.61
Venezuela	53.95	0.95	0.95	0.98	0.80	79.34	3.3	0.23	−0.24	−0.32	−0.02	−1.15	1.05
***Tropical Andes***	***64.04***	***0.89***	***0.92***	***0.95***	***0.73***	***44.10***	***2.06***	***0.23***	***−0.51***	***−0.24***	***−0.07***	***−1.36***	***0.86***
Burundi	19.67	0.96	0.95	0.97	0.88	100	29.13	0.51	−0.07	0.00	−0.10	0.00	0
D.R. Congo	71.18	0.97	0.95	0.98	0.96	55	3.25	0.12	−0.09	−0.27	−0.07	0.00	0.17
Ethiopia	14.27	0.96	0.92	0.98	0.91	24.55	6.76	0.58	−0.20	−0.21	−0.22	0.00	0.1
Kenya	7.47	0.97	0.95	0.98	0.95	54.60	5.71	1.2	−0.18	−0.23	−0.18	−0.10	0.27
Malawi	19.79	0.98	0.98	0.98	0.96	88.42	9.8	0.52	−0.13	−0.20	−0.14	0.00	0
Mozambique	43	0.98	0.97	0.98	0.98	19.70	3.71	0.57	−0.13	−0.17	−0.15	0.00	0
Rwanda	18.92	0.95	0.93	0.97	0.86	38.57	27.48	0.54	−0.10	−0.18	−0.09	0.00	0
South Sudan	22.18	0.99	0.97	0.99	1.00	64	1.39	0.39	−0.18	−0.32	−0.17	0.00	0.45
Tanzania	24.3	0.95	0.93	0.97	0.84	60.97	4.66	0.66	−0.14	−0.17	−0.11	−0.23	0.07
Uganda	21.45	0.98	0.96	0.99	0.95	61.61	8.24	0.45	−0.11	−0.18	−0.11	0.00	0.61
Zambia	38.52	0.98	0.97	0.99	1.00	59.66	2.09	0.47	−0.09	−0.16	−0.09	0.00	0.19
***Africa Great Lakes***	***25.07***	***0.96***	***0.94***	***0.98***	***0.93***	***62.89***	***7.15***	***0.40***	***−0.14***	***−0.27***	***−0.14***	***−0.05***	***0.20***
Cambodia	49.81	0.94	0.88	0.96	0.96	42.53	8.91	0.57	−0.57	−1.79	−0.37	−0.19	1.42
China	13.17	0.92	0.91	0.96	0.79	53.00	11.36	0.45	−0.42	−0.88	−0.14	−1.03	1.54
Lao P.D.R.	69.06	0.95	0.88	0.98	0.95	62.80	8.51	0.54	−0.40	−1.42	−0.21	−0.33	2.09
Myanmar	50.22	0.95	0.90	0.97	0.97	35.38	9.52	0.55	−0.54	−1.20	−0.47	−0.23	0.92
Thailand	31.77	0.95	0.89	0.96	0.96	73.41	9.37	0.81	−0.82	−1.57	−0.72	−0.56	1.19
Vietnam	38.27	0.94	0.87	0.96	0.91	33.68	19.44	0.4	−0.42	−1.45	−0.16	−0.77	0.99
***Greater Mekong***	***44.03***	***0.94***	***0.89***	***0.97***	***0.91***	***49.21***	***6.34***	***0.53***	***−0.46***	***−1.29***	***−0.19***	***−0.98***	***1.43***

Together, the dashboard graphics present a picture of the current status (Figure 5) and trends (Figure 6) in biodiversity. Baseline data show large geographical variation in the status of forest cover. National forest coverage is the lowest in Kenya (7.47%), and the highest in D. R. Congo (71.18%) and Lao P.D.R. (69.06%) (Table 3 and Figure 5A). The baseline Red List Index reveals high species extinction risk in Tropical Andes for all taxa (0.89), with variations among countries and among taxonomic groups (Table 3). The conservation response, measured as the average percentage of key biodiversity areas under protection, varies somewhat among regions (44% in the Tropical Andes, 63% in the African Great Lakes region, and 49% in the Greater Mekong as of 2010) but the dashboard shows larger differences at national levels, with lows in Mozambique (20%), Ethiopia (25%), Peru (25%), Vietnam (34%) and Myanmar (35%), and highs in Burundi (100%), Malawi (88%), Venezuela (79%) and Thailand (73%) (Table 3 and Figure 5C). Similarly, baseline data for freshwater provision show large national differences (Figure 5D), with Burundi, Rwanda, Vietnam, and China standing out as areas of high importance.

The trend of forest loss is documented as ongoing in all regions evaluated, with national rates of loss lowest in Peru (0.08%/yr) and D. R. Congo (0.12%/yr), and highest in Kenya (1.2%/yr) (Table 3 and Figure 6A). The Red List Index indicates the worsening status of species, with a decline in Red List Index observed for all nations between 1980 and 2008 (Table 2). Rates of decline were highest in the Tropical Andes, largely driven by amphibians (1.36×10^−3^/yr), and in the Mekong, due to both mammals (1.29×10^−3^/yr) and amphibians (0.98×10^−3^/yr). Protection of key biodiversity areas increased in all regions since 1980, with some regional variation in the rate of increase (0.20% in the African Great Lakes, 0.86% in the Tropical Andes, and 1.43% in the Greater Mekong). Nationally, rates of increase in the protection of key biodiversity areas ranged from lows of zero in Burundi, Malawi, Mozambique and Rwanda, and highs of 2.09%, 1.54%, and 1.42% in Lao P.D.R., China, and Cambodia (Table 3 and Figure 6C).

## Discussion

### 1. National and Regional Monitoring Challenges

As approaches to biodiversity management shift towards data-intensive and science-driven methods [74], [75], addressing gaps in capacity for information generation and dissemination has become increasingly important [76], [77], [78], [79], [80]. The prevailing and widely recognized challenges to addressing these gaps include sustaining financial and human resources for on-the-ground monitoring [76], [81], overcoming cultural and technical barriers associated with data generation [82], [83], [84] and information sharing [74], [85], [86], adopting scientific standards for monitoring and data analysis [79], [82], [84], [87], and developing indicator sets that can effectively inform policy and decision-making processes [10], [40], [88], [89]. Our survey of regional biodiversity experts reaffirms these challenges in generating, managing, and sharing biodiversity information (Figure 4), demonstrates the strong demand for access to biodiversity status and trend data at multiple spatial scales, and indicates that our proposed biodiversity indicators dashboard could be an effective tool to address varied conservation needs (Figure 3).

Recognizing that national and local indicator data are often limited or non-existent, the survey respondents affirmed the value of deriving indicators from global datasets as an intermediate measure necessary to meet current demand. Moving forward, the respondents noted that it will be necessary to augment and validate globally-derived measures with national and local monitoring results, and doing so will require both cost-effective participatory monitoring protocols that ensure sustainable data collection and well-designed standards that ensure data interoperability (Figure 4). A lack of baseline data was the most frequently mentioned monitoring challenge in our survey. The few studies that have systematically evaluated the availability of indicators for monitoring biodiversity targets [90], [91] support our findings that biodiversity indicators, particularly indicators of state and benefit, are deficient (Figure 2). Unstable political situations, lack of financial support, and the low priority of biodiversity monitoring culturally or in national development strategies all can impair continuous and systematic data collection. At the same time, the barriers to information access and interoperability, prevent the information that does exist from fully informing conservation efforts. Biodiversity data are generated and kept by different agencies in a fragmented manner. Within our area of study, civil society and academics (such as the Wildlife Conservation Society in Cambodia, NatureKenya in Kenya, NatureUganda in Uganda) accumulate a wide range of site-level monitoring data maintained as project-based resources, while national level monitoring data are typically held by specific government divisions who may or may not share that information with other government entities, much less outside organizations. These challenges are well-known and efforts to address them are being made at global (e.g., Biodiversity Indicators Partnership [12]), regional (e.g., ASEAN Centre for Biodiversity [92], Red Amazónica de Información Socioambiental Georreferenciada [93], Streamlining European Biodiversity Indicators [94], Conservation of Arctic Flora and Fauna [95]), and national levels (e.g., National Biodiversity Data bank in Uganda [96], National Biodiversity Database System in Vietnam). However, those efforts do not cover all countries and regions, and ready access to reliable and geographically consistent biodiversity indicator information at multiple scales remains a confirmed need.

### 2. Towards a Dashboard

The biodiversity indicators dashboard, as explained here, is designed to address the unmet needs expressed in our survey of biodiversity experts by laying the foundation for better accessibility and interpretability of existing biodiversity trend data within a framework that enhances monitoring capacity and promotes data interoperability and sharing. Many of these indicators are widely used at global scales, but until now have rarely been reported at national scales. While previous studies have demonstrated the utility of biodiversity indicators in monitoring conservation status and trends [14], [22], our biodiversity indicators dashboard is the first operationalized online interface that communicates multi-dimensional indicators with spatial representation. By providing easy access to indicator information at national and local scales, it complements global efforts such as the Biodiversity Indicators Partnership [12] and facilitates reporting for Aichi National Biodiversity Strategies and Action Plans (NBSAPs). The intuitive graphics and ease of data access that flow from the dashboard are intended to engage and enhance partnerships at all levels – international, regional, national, and local.

As a data visualization tool, the biodiversity indicators dashboard is designed so that a quick examination communicates the overall status of biodiversity conservation, important trends and patterns, and previously hidden challenges. For example, the graphics and values for the state indicator, such as those presented in Table 3 and Figure 7, communicate a high extinction risk in the Tropical Andes, driven largely by the extinction risk of amphibian species. This finding is consistent with other recent studies [97], [98] and highlights the importance of addressing threats to amphibian species if biodiversity is to be maintained.

By using data and methods that are globally consistent, the dashboard facilitates direct comparison of baseline and trends across regions and nations in the three continents targeted for this first stage of dashboard developments. Regional patterns are readily evident, such as the higher rate of decline of the Red List Index in the tropical Andes or the enormous importance of fresh water provisioning in the Greater Mekong countries (Figure 5). Stark differences in the efforts of neighboring countries at safeguarding key biodiversity areas, such as for Mozambique and Tanzania, are also easily discernible by a non-scientific audience (Figure 5).

To develop the dashboard concept, we used indicators derived from global data to bypass the many obstacles in obtaining consistent and comprehensive regional and national data. While the reporting of these global indicators at the national level provides new and valuable information, there are limitations in using global data to represent national indicators. While advances in remote sensing technology provide an unprecedented opportunity to gain temporally repetitive information [99], currently available estimates of land cover change can differ substantively among sources (i.e., FAO forest resource assessment [100], Global Forest Cover Loss mapping [48], ESA Global Land Cover and GlobCover products [101], [102]) and are often coarse in resolution (342 km^2^ and 2592 km^2^ respectively for the deforestation and freshwater provision datasets used here). With regard to protected areas, global datasets tend to omit recently decreed areas and often fail to capture important differences in on-the-ground management. Similarly, the use of global distributions and Red List categories may not adequately reflect the conservation status of a given species in a particular region, and because global distribution data is only available for terrestrial vertebrates, the results do not reflect the status of many other components of biodiversity.

The shortcomings of indicators derived from global data can be addressed by integrating nationally and locally derived data into the final dashboard design. Despite the numerous obstacles, data are being generated at a variety of scales deemed useful by survey respondents, both through governmental efforts [92], [96], [103], [104], regional consortiums [92], [93], [94], [105], [106], [107], and site-specific projects [108], [109]. The next stage of the biodiversity indicators dashboard development focuses on building an effective data sharing mechanism the promotes shared identifiers to link data from these different sources [83], digital architecture to coordinate data flow and ensure data ownership, and promoting consent and trust among data contributors. With continued development, we envision the biodiversity indicators dashboard as an interactive, web-accessible platform that can facilitate national reporting towards biodiversity targets while allowing for the integration of localized data to support the type of site-scale monitoring deemed important to survey participants. The dashboard framework has also been designed so that over time, the indicators discussed here can be supplemented with other metrics capturing complementary aspects of each of state, pressures, responses and benefits (e.g. population trends, agricultural intensity, environmental legislation and additional ecosystem service measures).

By serving these data as a web-accessible dashboard, we can put information on status and trends in biodiversity within easy access of users and organizations from all sectors and backgrounds, and facilitate more informed decision-making, enable exploration of patterns among variables, and support the tracking of progress towards conservation goals. The maps presented here and information contained in Table 3 can be depicted in a dashboard format via various means, including as mapped values (Figures 5 and 6), mouse-over boxes displaying the numerical values associated with those maps, tabular data summaries by nation or indicator accessible via interactive menus, and charts of trends that users could generate either by region (Figure 7) or nation.

In agreeing to the Aichi Targets, the nations of the world implicitly committed to developing the data necessary to effectively monitor progress towards meeting biodiversity goals. The challenges in reporting towards those goals are many, but we believe the dashboard approach, as outlined here, provides a valuable framework that can facilitate and advance the type of reporting required by the conservation community. Starting with global indicators and expanding to incorporate additional national and site-scale data identified as important by conservation practitioners on the ground, the biodiversity indicators dashboard can serve as a tool to track progress towards Aichi Targets, support national monitoring and reporting, and inform outcome-based policy-making in the realm of conservation.
